# Combating infectious disease epidemics through China’s Belt and Road Initiative

**DOI:** 10.1371/journal.pntd.0007107

**Published:** 2019-04-18

**Authors:** Jin Chen, Robert Bergquist, Xiao-Nong Zhou, Jing-Bo Xue, Men-Bao Qian

**Affiliations:** 1 National Institute of Parasitic Diseases, Chinese Center for Disease Control and Prevention, Shanghai, China; 2 Chinese Center for Tropical Diseases Research, Shanghai, China; 3 WHO Collaborating Centre for Tropical Diseases, Shanghai, China; 4 National Center for International Research on Tropical Diseases, Shanghai, China; 5 Key Laboratory of Parasite and Vector Biology, Ministry of Health, Shanghai, China; 6 Ingerod, Brastad, Sweden; Jiangsu Institute of Parasitic Diseases, CHINA

## Introduction

On March 17, 2017, the United Nations (UN) Security Council, backed by the consensus of its 193 member states, adopted Resolution 2344, which calls for strengthening regional economic cooperation through China’s Belt and Road (also called Silk Road) Initiative (BRI) [1]. Based on the cornerstones of peace and cooperation, openness and inclusiveness, and mutual learning and sharing benefit, this initiative was proposed by the Chinese government and is participated in by various parties. Its goal is to provide fundamental solutions to boost global economic development through enhancing policy coordination, facility connection, unimpeded trade, financial integration, and people-to-people bonds. The UN Secretary-General, Mr. António Guterres, acknowledged that the BRI tallies with, and complements, the Sustainable Development Goals (SDGs) in terms of promoting inclusive development, strengthening exchange between countries, and benefiting people within the initiative’s scope [2]. The BRI currently involves 69 countries (mainly those named in the initial Belt and Road outreach and those having signed cooperation agreements), 70% of the world population in total, 30% of the global gross domestic product, and 75% of the world's energy reserves [2–4].

Although the BRI is primarily economic, it also includes important health dimensions. The Healthy China 2030 plan, promulgated in 2016, considers health as one of the national policy priorities, and the Memorandum of Understanding with the World Health Organization (WHO), signed in 2017, which promotes global health security and development along the terms of the initiative [5, 6]. Based on these agreements, the building of a Health Silk Road has become a core task leading to an extensive engagement in global health development [7, 8]. During the Belt and Road High-Level Meeting for Health Cooperation: Towards a Health Silk Road, held in August 2017 in Beijing, the Director-General of WHO, Dr Tedros Adhanom Ghebreyesus, positively commented on the initiative, saying that it may become the stimulus needed to drive our united activities towards universal health coverage (UHC) and that it contains the necessary fundamentals, such as infrastructure building, access to medicines, and human resources, needed to build a platform for sharing experience and promoting best practices [5]. The Health Silk Road concept establishes the importance of promoting cooperation among the related countries in the prevention and control of communicable diseases, medical system and policies, healthcare capacity building, staff training and exchange, traditional medicine, and health education as well as disaster relief, aid, and poverty reduction for health [1]. In this way, building the contextual Health Silk Road would advance political commitment to mobilise all resources for better health in the world.

The world is currently witnessing increasingly complex epidemics as well as natural disasters with a rising impact on both human health and the economy [1]. Growing commercial trade and more frequent personal exchange following the implementation of the BRI may amplify infectious disease transmission or inadvertently introduce emerging infectious diseases, leading to an increased burden for local medical systems. Moreover, infectious diseases resulting from poverty disproportionately affect poor and marginalised communities, which remains a more serious hurdle to achieving the SDGs and UHC [9, 10]. This is the reason why the initiative has made the need to combat infectious diseases a priority for social and economic development. The implementation of the BRI is expected to facilitate progress in eliminating infectious diseases such as the acquired immunodeficiency syndrome (AIDS) caused by the human immunodeficiency virus (HIV), tuberculosis (TB), malaria, and 17 neglected tropical diseases (NTDs), which make up SDG 3.3. It has also gained the support of WHO, which has proposed a strategic cooperation with the BRI [5, 6, 11]. Taking the opportunities provided by this initiative, action for combating the diseases will be conducted through the sharing of information and experience and cooperative disease control programmes, including interventions and research innovation [5]. It will also mediate resources including the building of medical infrastructures, funding support, training of staff, and delivery of emergency healthcare [1, 10, 12]. The initiative provides guidance for stronger health collaboration, which should hopefully break the vicious cycle of poverty and infectious disease [13].

Delegates from the Joint UN Programme on HIV/AIDS (UNAIDS), the Global Fund (https://www.theglobalfund.org/en/), the Global Alliance for Vaccines and Immunisation (GAVI) (https://www.gavi.org/), and more than 30 countries participated in the Belt and Road High-Level Meeting for Health Cooperation, signed the Beijing Communiqué, and agreed to participate in building the Health Silk Road. As for the infectious diseases, cooperation on control and elimination has been the highest priority, together with mounting an effective response to disease outbreaks and reemerging infections [2]. However, details regarding how this should be carried out in practice have not yet been presented; in particular, we still lack proposals that take up the challenges, specify indicators of expected impact, and develop opportunities for collaboration in this area. The purpose of this paper is to analyse the existing spectrum of the main infectious diseases in epidemics (based on the Global Burden of Diseases [GBD] study of 2016 [14, 15]), the potential negative effect on the economy, and the challenges to elimination. The paper also aims to explore opportunities and a feasible approach of global disease control with specific consideration of the BRI.

## Challenges regarding infectious diseases in Belt and Road countries

### The spectrum of local infectious diseases

The thrust of the BRI has two main directions: the Silk Road, which connects China by land with European countries via central and western Asia and the Middle East, and the 21st Century Maritime connection across the South China Sea, the Indian Ocean, and the Mediterranean to reach sub-Saharan Africa, following the lead of the great Chinese Ming dynasty admiral and explorer Zheng He. These two land and maritime directions from China follow five routes [1].

The Health Silk Road, a core task of the BRI, encourages health development and security along the Road. The 2030 Agenda for SDGs also calls for halting HIV/AIDS, TB, and malaria as well as the termination of the ravages caused by the 17 NTDs by 2030 [13]. Altogether, according to the 2016 GBD study, the cost expressed in disability-adjusted life years (DALYs) amounts to an estimated 63.31 million (with substantial regional variations in the 69 countries), which currently accounts for 36.7% of the total toll of these diseases throughout the world [2, 14, 15].

**Fig 1:**
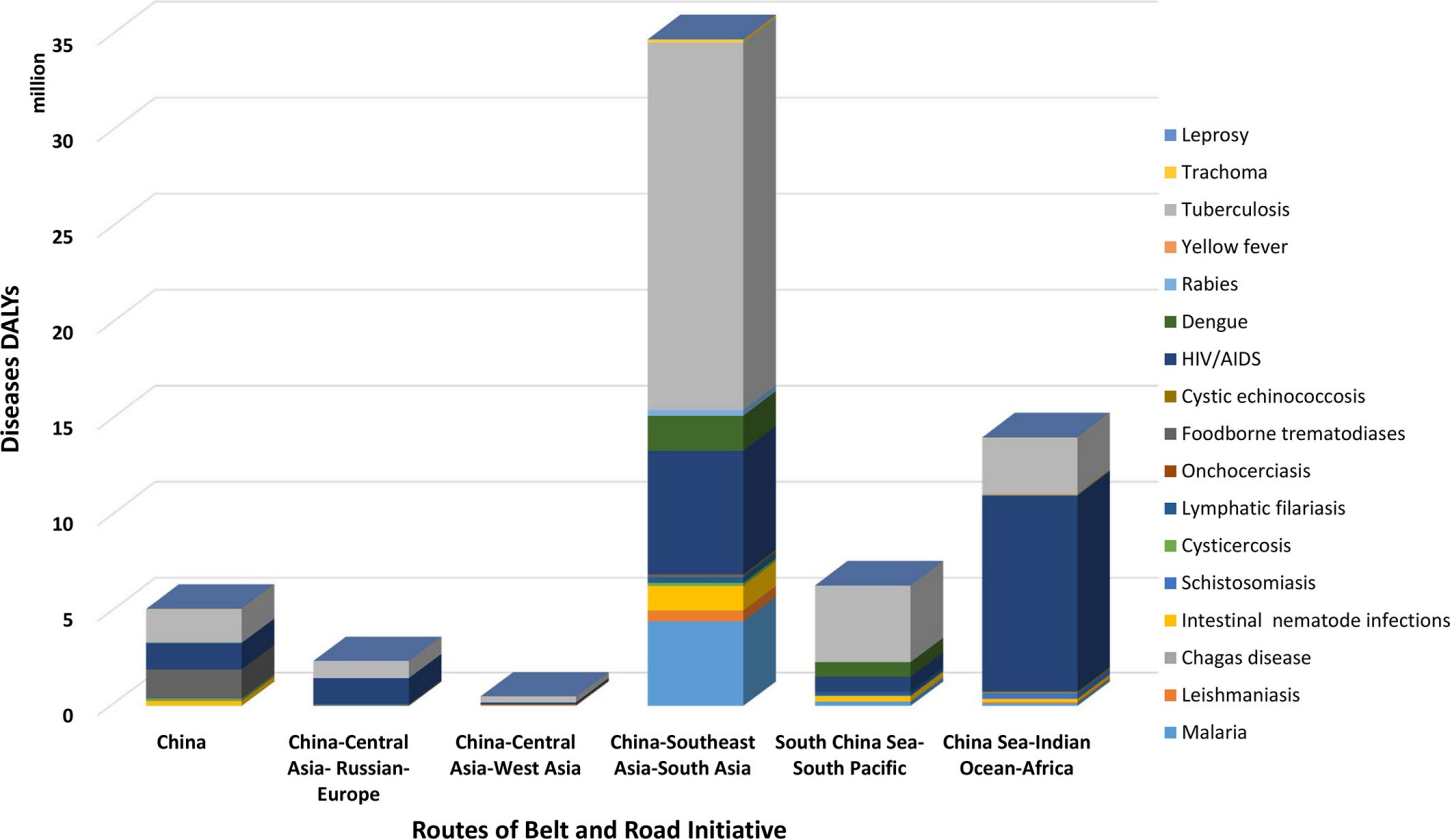
The DALYs of 20 infectious diseases along five routes of the Belt and Road Initiative. AIDS, acquired immunodeficiency syndrome; DALY, disability-adjusted life year; HIV, human immunodeficiency virus.

The DALYs resulting from the ‘big three’ (HIV/AIDS, TB, and malaria) plus those due to the 17 NTDs along the route from China towards Southeast Asia and South Asia (excluding China) amounts to 34.81 million DALYs and are mainly due to HIV/AIDS, TB, and malaria in addition to dengue and intestinal nematode infections. The infectious disease epidemics in countries along the 21st Century Maritime Silk Road are mainly the same set of diseases plus schistosomiasis, which together generate 20.46 million DALYs. The China–central Asia–Russia–Europe route, involving countries in central Asia and eastern Europe, includes many of the same diseases as well as some additional ones—i.e., intestinal nematode infections, cystic echinococcosis, foodborne trematodiases, and cysticercosis—which together cause 2.39 million DALYs. A total of 0.50 million DALYs in the countries along the China–central Asia–west Asia axis are due to TB, leishmaniasis, malaria, HIV/AIDS, intestinal nematode infections, and cystic echinococcosis (Fig 1). Such high endemicity of infectious diseases in the region is a serious hurdle for the global elimination agenda. The DALYs due to these diseases vary across the regions, and so do the collaborative priorities and their expected potential impacts. For example, cooperative actions could lead to regional elimination of lymphatic filariasis (LF) along the Maritime Silk Road while simultaneously having a significant impact on malaria, dengue, intestinal helminth infections, schistosomiasis, and TB [16, 17].

### Disease-specific consideration for cooperation

**Fig 2:**
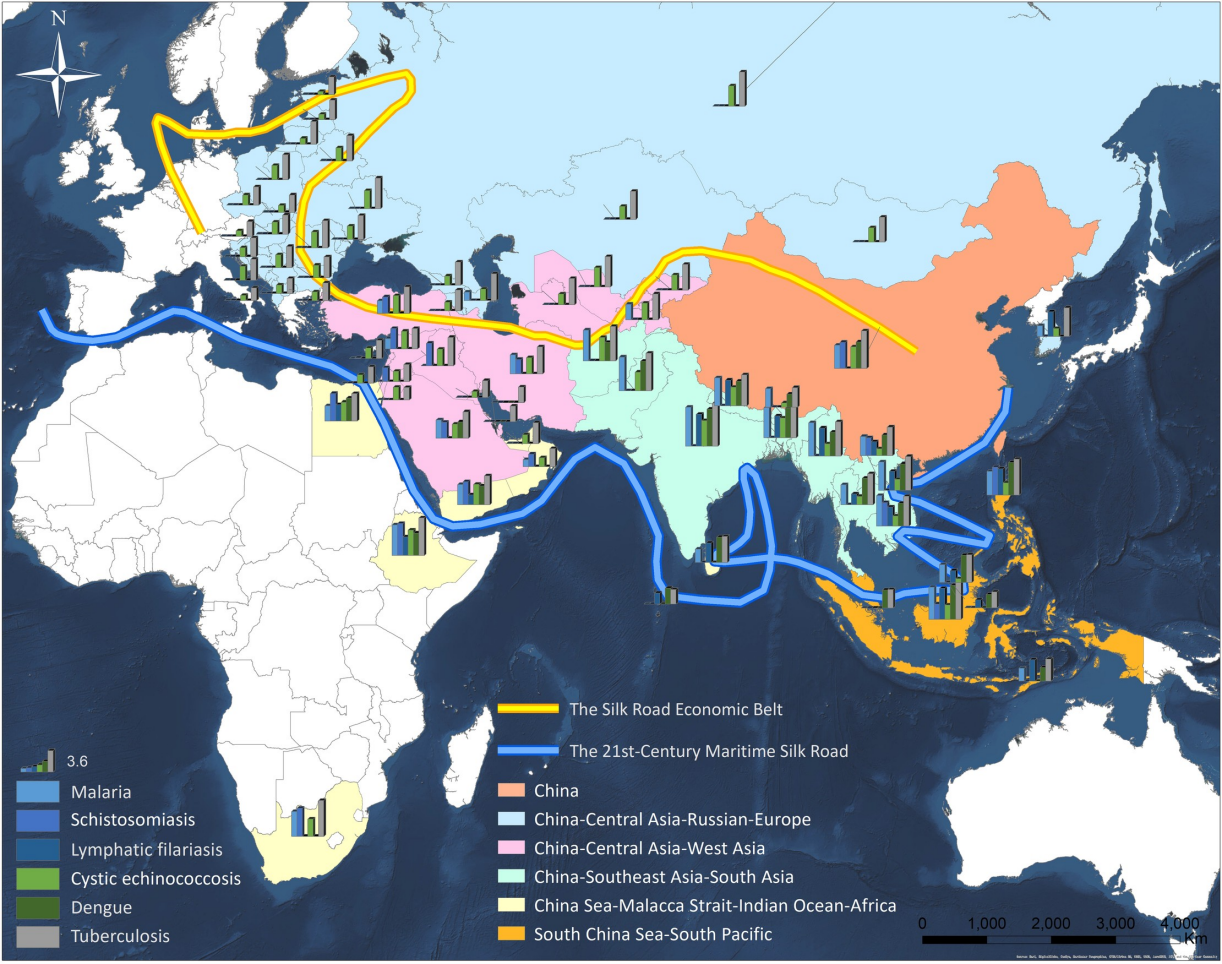
The spectrum of six infectious diseases in the Belt and Road countries. The value of DALYs data has been processed through the logarithmic function. DALY, disability-adjusted life year.

Considering the high number of DALYs caused by the previously mentioned 20 diseases, their regional distributions and cross-border transmission risks, and the experience combating these diseases, six of them (malaria, schistosomiasis, LF, TB, dengue, and cystic echinococcosis) have been listed for collaborative control and elimination as priorities in the Belt and Road Work Plan for the Health Silk Road [1, 18]. Together, these six diseases cause a considerable disease burden in these countries, impeding economic growth and constituting a serious challenge with regard to elimination (Fig 2) [14]. For example, dengue causes 2.65 million DALYs, which accounts for 89.7% of this disease's global disease burden [14]; TB inflicts an estimated 29 million DALYs, which represents 66.7% of its total DALYs score [12]; cystic echinococcosis causes 0.07 million DALYs (half of the total number in the world); the current DALYs score of LF reaches 0.47 million, accounting for 39.7% of the global count [14]; and malaria and schistosomiasis generate an estimated 4.87 million and 0.39 million DALYs, respectively, in the Belt and Road countries.

Patients infected with these diseases not only suffer from disability but also have to endure expenses for treatment and productivity disruption. In addition to individual suffering, these diseases hinder economic development. TB, for example, adds a significant economic burden that is difficult to estimate exactly, as it impedes development both at the national and individual-household levels. This disease has a higher prevalence in the economically unprivileged sector of the population, and the cost for healthcare, including diagnostics, accommodation, transport, and ancillary drugs, is high [19, 20]. Annual costs for cystic echinococcosis, on the other hand, have been estimated at USD 3 billion, which reflects that the treatment is expensive not only because it requires human treatment but also because it requires health education, improved slaughterhouse hygiene, and deworming of the local dog population [21, 22]. All of these diseases lead directly to loss of productivity, which worsens the situation of vulnerable populations, in turn resulting in continued poverty [23, 24]. In addition, they affect agricultural, fishing, and pastoral populations in poor regions, while the ongoing influx of people to urban areas (including tourism) increases the risk of disease expansion into new areas.

Although much has been achieved, substantial gaps to controlling and eliminating these six diseases remain, especially with increasing communication and trade across borders of these countries. Drug resistance and coinfection result in additional difficulties in conducting control programmes, e.g., the rising mosquito resistance to insecticides as well as the parasites' ability to develop drug resistance. As strategies aimed at TB elimination are strongly challenged by multidrug-resistant bacterial strains (MDR-TB) [11, 25], the estimated mortality from TB in people living with HIV was as high as 30% (0.4 million/1.4 million) in 2015 [26]. Imported cases represent a threat for the countries that are close to accomplishing elimination, and prevention and control of dengue, malaria, schistosomiasis, and LF demand a well-developed surveillance and response system with regard to vector management and sustainable interventions at all levels [27, 28]. Control of schistosomiasis and cystic echinococcosis require integrated approaches from the agriculture and husbandry department because of their zoonotic characteristics.

Infectious diseases not only cause loss of health and, eventually, life but generally also lead to economic consequences. Serious challenges oppose current efforts to stem and limit epidemics in these countries, requiring urgent collaboration with adjacent and global countries through shared experience, financial support, multisector intervention, professional healthcare delivery, and technical innovation.

## Collaborative opportunities when dealing with infectious diseases

Promoted by the BRI, with the Health Silk Road as a priority, opportunities will be provided for combating infectious disease epidemics based on technical experience communication, financial investment, cooperative organisation, and future collaborative programmes.

### Enhancing technical experience communication

Appropriate management and treatment are essential to achieving the elimination of infectious diseases [12]. China’s disease interventions have shown that control and elimination are feasible and can be carried out in a cost-effective way. However, such efforts will have to be tailored to local settings. In 2007, China successfully passed the validation of its LF elimination strategy after having followed a plan of control and surveillance for nearly 60 years [29]. The work towards elimination of LF in China has relied on the implementation of three schemes: (1) surveys using repeated blood testing followed by treatment when needed; (2) mass drug administration (MDA) with diethylcarbamazine-fortified salt to disrupt infectious microfilaria; and (3) target treatment of carriers using the same drug. Cambodia has improved its healthcare capacity and also managed to eliminate LF as a public health problem using a similar approach along with parallel interventions against other vector-borne diseases [30].

Turkmenistan and the United Arab Emirates have achieved zero indigenous cases of malaria [31], whereas China had no indigenous cases in 2017 [32]. The Chinese government has scheduled the elimination of this disease by 2020 [33]. As for the national malaria elimination programme, the 1-3-7 surveillance and response strategy (reporting cases within one day, investigating cases within three days, and targeting control measures within seven days) has benefitted from conducting interventions at the provincial, municipal, and county levels, ensuring a nationwide effect [34]. This successful rapid response to cases of malaria in returning overseas workers and the engagement of private-sector employers have recently been implemented in other Asia-Pacific countries [34]. Using the antimalarial drug artemisinin, developed from herb-based Chinese traditional medicine, has proved vital for malaria treatment [35]. In addition, the use of mosquito nets is suggested for implementation during the transmission season [29]. Schistosomiasis has been effectively controlled, with 90% of the previously endemic counties now free of the disease. The Chinese government now schedules schistosomiasis elimination by 2025 [36] by using the successful, multisectoral national strategy that has reduced *Schistosoma japonicum* infection in cattle, humans, and snails, which is based on MDA with praziquantel for snails combined with the removal of cattle from the grasslands where the snails reside [37].

With regard to TB, dengue, and cystic echinococcosis, the plan is to end these epidemics, and there has already been success in reaching this goal. For example, the estimated DALYs score for TB in China has dropped from 6.29 million to 1.74 million in the past 26 years (1990–2016) [14], which is attributed to the nationwide TB control programme using the directly observed treatment short-course (DOTS) approach [38]. The well-established, web-based communicable disease notification system incorporates the notification of new cases [26], and the national insurance scheme, a public–private partnership (PPP) tailored to support people with low incomes, covers more than 95% of the population [26, 39] and functions well. However, the effective strategy to end TB also requires high-level political commitment, community engagement, social protection, funding support, and innovation of drug delivery and rapid diagnostics.

For dengue, the timely reporting of cases also relies on a notification system, and the approach to controlling mosquito reproduction has provided a solution to dengue epidemics elsewhere [40]. Most clinicians agree on the four options for treating cystic echinococcosis: drugs, percutaneous methods, surgery, and ‘watch and wait’ [22]. In China, the control and treatment of echinococcosis integrates large-scale surveys, control of the transmission source (deworming definitive hosts), and detection plus treatment of patients free of charge, supported by domestic funding [41].

The successful interventions in China are implemented in line with the Chinese health system, which is vital for delivering healthcare to less developed regions and responds effectively to health crises by coordinated action [12]. Apart from the above interventions, political support, regulation guidance, and financial investment are paramount. It has been proposed by WHO to tailor some feasible aspects to local health systems to achieve disease elimination and UHC [42].

### Proceeding platforms for collaboration

The Health Silk Road concept encourages regional cooperation and extensive participation from governments, international and regional organisations, universities, private sectors, civil society, and the general public. China is a positive initiator and has, together with related countries, jointly published a series of agreements. Furthermore, 41 programmes are active, including the China–Association of Southeast Asian Nations (ASEAN) plan Training One Hundred Health Professionals and the China–Africa Cooperation Plan on Public Health. Nongovernmental exchange and cooperation activities in various fields are complementary to governmental projects, as they build public support for the BRI. China has also worked to strengthen think-tank cooperation and initiated the International Silk Road Think Tank Association [1].

Building the Silk Road for health by combating infectious diseases cannot be achieved without multisector engagement and expert involvement. The platforms created are well suited to take on current opportunities in collaboration with organisations such as WHO collaborating centres and existing South–South networks, linking them with similar units in Belt and Road countries (Table 1). Current platforms have significantly contributed to achieving first control and later elimination of some diseases and are now turning to the global level, providing opportunities to assist building a health approach within the BRI. Besides establishing platforms for combating diseases, China’s participation in control programmes within Asia and Africa has become a model for other countries [43]. For example, trilateral collaboration between China (providing technical support), Australia (a major donor), and Papua New Guinea (a country close to holoendemic for malaria) is dedicated to improving local staff capacity for malaria diagnosis and disease control. This programme highlights the regional health security agenda of a malaria-free Asia Pacific by 2030. In Africa, in the pilot China–United Kingdom–Tanzania malaria control programme, the parasite prevalence has dropped by two-thirds after intervention for three years, and Tanzania has established a ‘diagnosis–treatment–follow up’ prevention plan for malaria with links to China’s experience and WHO guidelines.

**Table 1:** Platforms for combating infectious disease epidemics.

Type	Platform Title	Participating Institution	Activity	
**WHO Collaborating Centres in China**	WHO Collaborating Centre for Tropical Diseases	National Institute of Parasitic Diseases, China CDC, Shanghai	Technical WHO advisory function; research on tropical diseases; coordination of activities	
	WHO Collaborating Centre for Research and Training on Tuberculosis	Beijing Tuberculosis and Thoracic Tumor Research Institute; National Center for Tuberculosis Control and Clinical Medicine, China CDC, Beijing	Technical WHO advisory function; training and education	
	WHO Collaborating Centre on Schistosomiasis Control in the Lake Regions	Hunan Institute of Parasitic Diseases, Yueyang, Hunan	Development and application of appropriate technology; research on parasitic diseases; training and education	
	WHO Collaborating Centre for Comprehensive Management of HIV Treatment and Care	STD/AIDS Centre for Treatment and Care, Beijing Ditan Hospital, Beijing	Information dissemination; organisation of events; training and education	
	WHO Collaborating Centre for Vector Surveillance and Management	National Institute for Communicable Disease Control and Prevention, China CDC, Beijing	Training and education; evaluation; product development	
	Collaborating Centre for Prevention and Care Management of Echinococcosis	Xinjiang Key Laboratory of Echinococcosis, Clinical Medical Research Institute, First Affiliated Hospital of Xinjiang Medical University, Xinjiang	Training and education; research on parasitic diseases; development and application of appropriate technology	
	WHO Collaborating Centre for Research and Training on Malaria Elimination	Jiangsu Institute of Parasitic Diseases, Wuxi, Jiangsu	Development and application of appropriate technology; research; training and education	
	WHO Collaborating Centre for Lymphatic Filariasis and Taeniasis/Cysticercosis	Shandong Institute of Parasitic Diseases, Jining, Shandong	Research; development and application of appropriate technology; implementation of WHO programmes and activities at the country level	
**South–South Networks on Tropical Diseases**	INCAS	Department of Control of Neglected Tropical Diseases, WHO; China CDC; some African countries	Share experience on schistosomiasis control and elimination; establish a research collaboration network between Africa and China on schistosomiasis control and elimination	
	RNAS^+^ (www.rnas.org.cn)	National Parasitic Institutes in Cambodia, China, Indonesia, Laos, Myanmar, the Philippines, Vietnam	Research and control of schistosomiasis and soil-transmitted helminth diseases in Southeast Asia	
	AP-NDI (www.apndi.org.cn)	Scientists from Australia, Cambodia, Canada, China, India, Laos, New Zealand, Singapore, South Korea, Thailand, United States of America	Share experience and promote research collaboration between Asia-Pacific research institutions	
**Regional Collaborations**	National Centre for Tropical Diseases, China, and other tropical-disease institutions and health centres	Ifakara Health Institute, Tanzania, Swiss Tropical and Public Health Institute, London School of Tropical Diseases and Public Health, Theodore Biharz Institute, Egypt; Sultan Blue Nile, Duke University, United States of America		Promote experience and knowledge translation
	Chinese Society of Global Health under Chinese Preventive Medical Association	Chinese Preventive Medical Association; National Institute of Parasitic Diseases, China CDC	Provide intellectual and human resources support	
	Belt and Road Network for Elimination and Control of Echinococcosis and Cysticercosis	Health-related programmes sponsored by governments, universities, NGOs, donors, and the private sectors from 13 countries	Mobilise available resources and involve multiple stakeholders for cestode zoonoses control	

Abbreviations: AD-NDI, Asia-Pacific Network for Tropical Medicine Diagnosis and Innovation; China CDC, Chinese Center for Disease Control and Prevention; INCAS, Institution-based Network on China-Africa Cooperation for Schistosomiasis Control; NGO, nongovernmental organization; RNAS^+^, Regional Network for Asian Schistosomiasis and Other Helminth Zoonoses; STD, sexually transmitted disease.

The Regional Network for Asian Schistosomiasis and Other Helminth Zoonoses (RNAS^+^) has contributed to sharing experience and promoting control of helminth infections in Asia for the past 20 years. The Belt and Road Network for Elimination and Control of Echinococcosis and Cysticercosis, involving institutions from Asia and Europe, has established the importance of technical cooperation and health education, and regional collaborating control activities are on the way. In the 2018–2022 Five-Year Plan of Action on Lancang–Mekong Cooperation, infectious disease control is achieved by strengthening collaboration on dengue fever and malaria and establishing and improving the mechanism for joint surveillance, prevention, and control of cross-border emerging and reemerging infectious diseases. Other programmes, such as the Cooperation in Control of Infectious Diseases in Central Asia, cooperates with central Asian countries on the control of echinococcosis and other zoonoses, and LF and TB control are being proposed to enlarge, enforce, and deepen cooperation worldwide.

Regional cooperation will be increased through health infrastructure work by building joint laboratories and research and knowledge translation centres [44]. China will upgrade 50 medical and health aid programmes for Africa, such as the headquarters of the African Center for Disease Control and Prevention and China–Africa Friendship Hospitals [45]. China has also donated drugs and equipment as well as helped to build health facilities [35]. The BRI will also involve improved access to healthcare by China’s innovative private sectors. For example, responding to the Ebola epidemic, the Aucma Company (Qingdao, China) has facilitated the delivery of the Ebola vaccine to West Africa using the Arktek (Qingdao, China) portable cold-storage devices. The diverse and vibrant private sectors in China, such as those dealing with data systems and mobile phone–based technologies, will potentially provide much-needed assistance for combating infectious diseases [46]. In this collaboration based on the principle of ‘joint construction and mutual benefit', the programmes employ local medical staff to carry out evidence-based studies and deliver healthcare and treatment to local people. This not only provides technical guidance, establishes the mechanism for disease control and prevention, and improves local staff capacity to sustain interventions but also produces open platforms and innovative new models for global health collaboration.

### Financial and human resource support

Ending infectious disease epidemics demands intensified funding, preferably delivered at the international level, to strengthen advocacy, research, and the global control effort. Besides being a sincere collaborator in the area of global health and a board member of UNAIDS and the Global Fund [11], China is creating its own multilateral funds and banks, e.g., the Asia Infrastructure Investment Bank and the New Development Bank, to promote regional cooperation in the framework of the BRI. The multilateral funds are substantial, adding up to USD 267 billion, with China securing major pledges, mainly from the two banks and the Silk Road Fund. Part of the financial aid is supposed to contribute to research and innovation with respect to medical products and diagnostic skills. The initiative also encourages discovery and production of new drugs and vaccines [4, 44]. Specifically, a total of USD 2 billion has gone to the South–South Cooperation Trust Fund to support developing countries in implementing the SDGs, and a further fund of USD 2 billion (to be increased to USD 12 billion by 2030) will assist many developing countries to meet the SDGs. Also, an additional voluntary contribution of USD 20 million will be provided to support WHO’s global health work, especially on infectious diseases [11].

The initiative is also working on poverty reduction to alleviate the epidemics—for example, the East Asia Poverty Reduction, Pilot China–Africa Cooperation Plan for Poverty Reduction, and People’s Benefit programmes, which provide aid in the fields of poverty reduction and healthcare. In addition, China will cancel the debt of the least developed countries, launch 600 specific projects to end poverty, and support better health services [11]. In this way it can alleviate regional poverty so that the income of local people will increase and their living standards will improve, and it can reduce the risk and burden of infectious diseases caused by poverty.

Apart from funding infrastructure construction, which supplies delivery and research, the BRI also advocates improved professional and organisational capabilities targeting specific diseases [4]. As one of the WHO Emergency Medical Teams, China has distributed medical supplies and will, together with WHO, take on infectious disease control, implementing professional support, training of medical staff, and healthcare delivery. For instance, during the Ebola pandemic, China sent 1,200 medical experts and committed an additional USD 120 million for training more than 13,000 local medical workers in response to Ebola infections in West Africa [11]. China will assist in training more medical personnel for Africa and continue to send medical teams to meet Africa's requirements [45]. More global health human resources and specific medical workers will be brought in, together with global health departments being established in universities and institutions [47].

China is pursuing discussions about cooperation with multilateral initiatives at the highest level, aligning relevant health agreements under the BRI theme. Compared with other initiatives, this initiative stands out because of its political commitment, which advances the health SDGs and solves current constraints to the progress of UHC [5, 48]. Chinese channels of funding and expenditure on health focus are different from those in the United States when it comes to funding in that the US focuses on specific diseases like HIV/AIDS, malaria, and TB through bilateral and multilateral channels, whereas China mainly focuses on medical facilities, health supplies, technical support, and training through governmental funding. However, the situation is changing, as the Belt and Road multilateral funding initiative is substantial, with a total planned to reach USD 267 billion, exceeding the USD 253 billion capitalisation of the World Bank [4].

As an ambitious, long-term initiative that is extremely open and inclusive, the BRI meets the great challenges of cultural differences and economic risks and has questioned the viability of some infrastructure investments. However, with the goal of a shared benefit for all humankind, an increasing number of countries have engaged in building the initiative. Enhanced interconnectivity will bring about not only international health risks but also, more importantly, new opportunities for global health cooperation and development. So far there is a lack of specific indicators for each disease and detailed procedures regarding cooperation with respect to disease control and elimination. The current study is expected to result in recommendations to policy makers on an overall policy plan and a road map for the imminent future.

All aforementioned experiences, platforms, and programmes as well as financial and personnel resources provide useful opportunities for cooperation among involved countries with respect to the threat of infectious diseases. More importantly, pilot research and demonstration projects in line with the BRI have been proposed and, in some cases, have already started. China has demonstrated strong and sustained political leadership to ensure its core role of global health collaboration in economic development.

## Conclusion and implication

**Fig 3:**
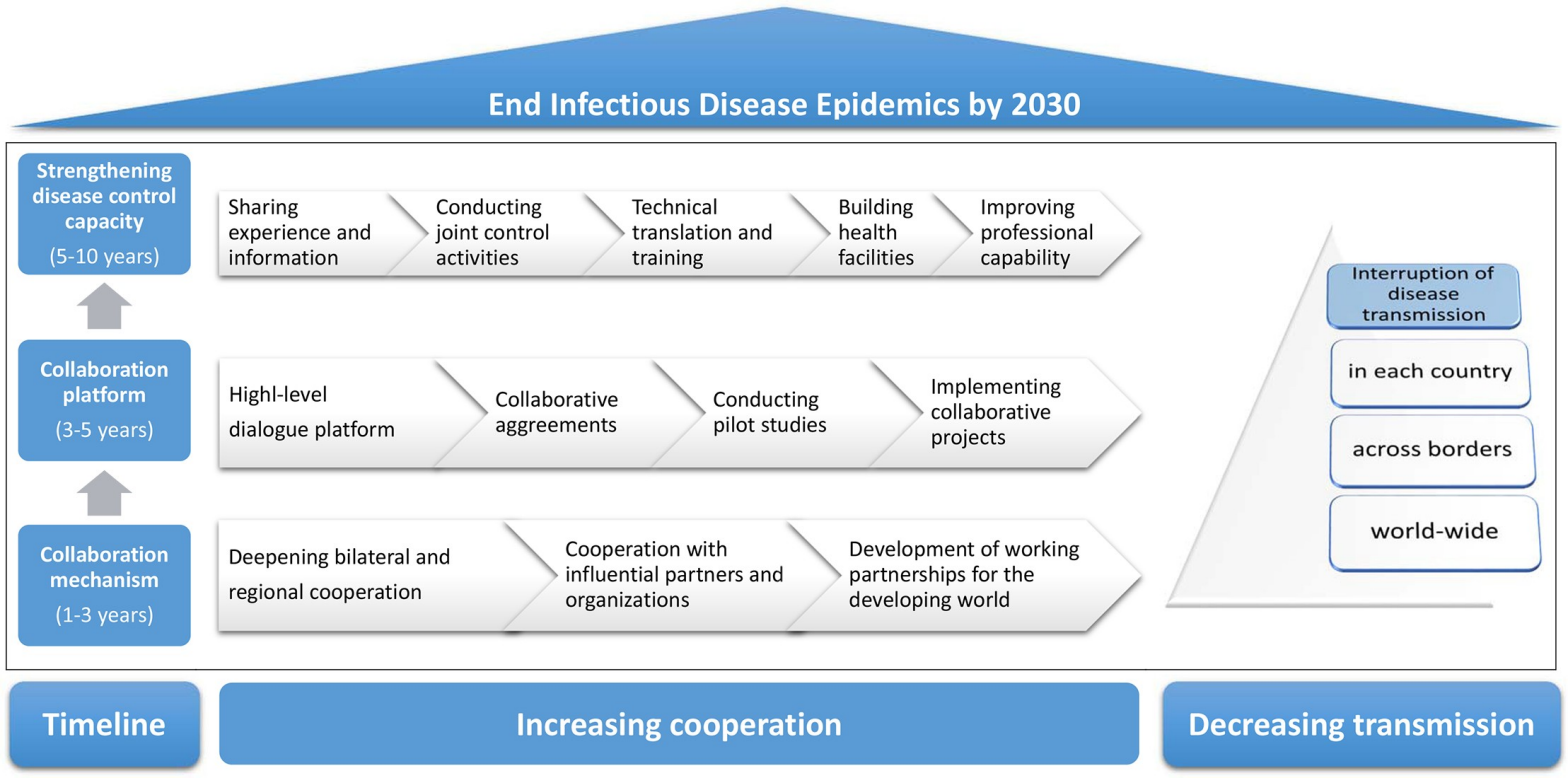
The road map of combating infectious disease epidemics.

A major part of the BRI focuses on support and communication to build a new mechanism for global health, prioritising the prevention and control of infectious diseases, preventing outbreaks becoming epidemics, and providing UHC, thus overcoming the vicious circle of poverty and ill health. China will strengthen cooperation with particular regard to the control of TB, echinococcosis, and dengue within the 69 countries and deliver enhanced communication and research leading to the elimination of LF, malaria, and schistosomiasis. Based on opportunities the BRI provides and the cooperative experience gained, the framework shown in Fig 3 should become available and applicable to the response to these challenges by sharing information, joint control, and technical know-how.

UHC and response to the challenges posed by infectious disease epidemics are vital for the new era, with health considerations at the core of the BRI. Despite the serious threats of the infectious disease epidemics, the emphasis on health through the BRI puts us in an excellent position to achieve the health-related aspects of the SDGs by implementing the Health Silk Road concept of improved life through health-related communication. Based on technical experience in this field, mature collaborating mechanisms, and the provision of financial support, the strategies in the context of the BRI reinforce the various countries’ extensive engagement in combating infectious disease epidemics.
